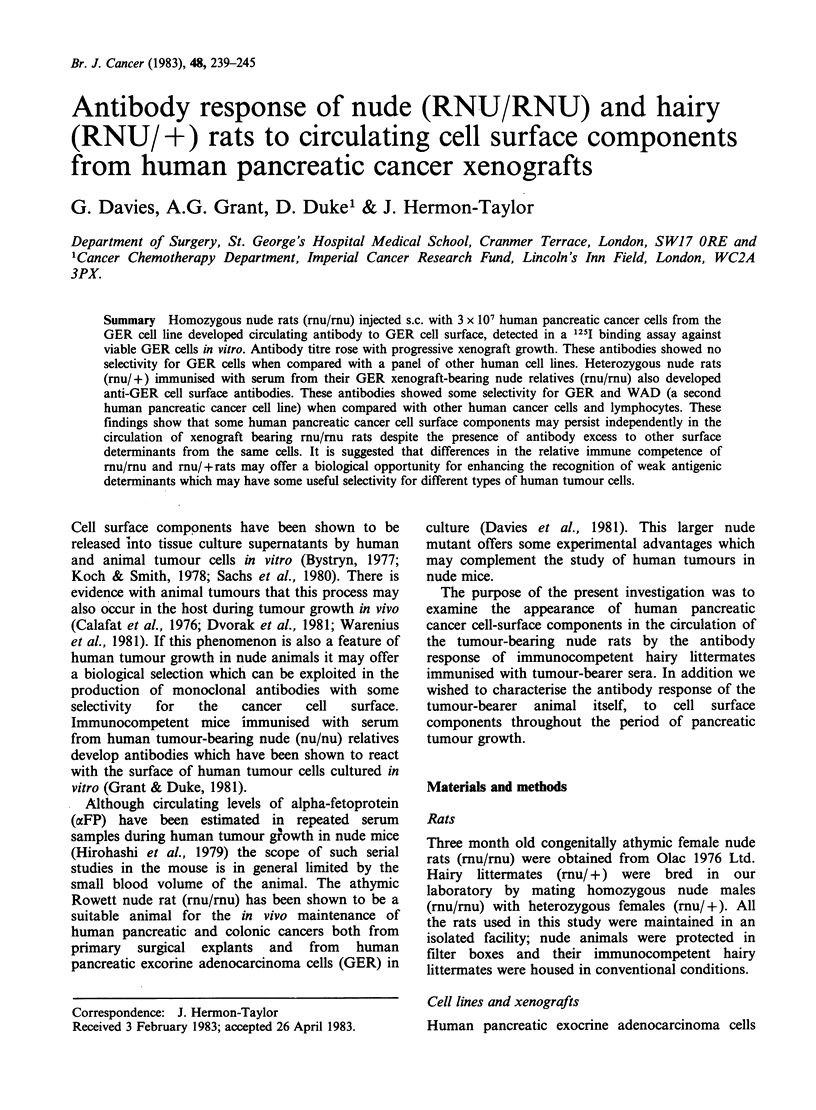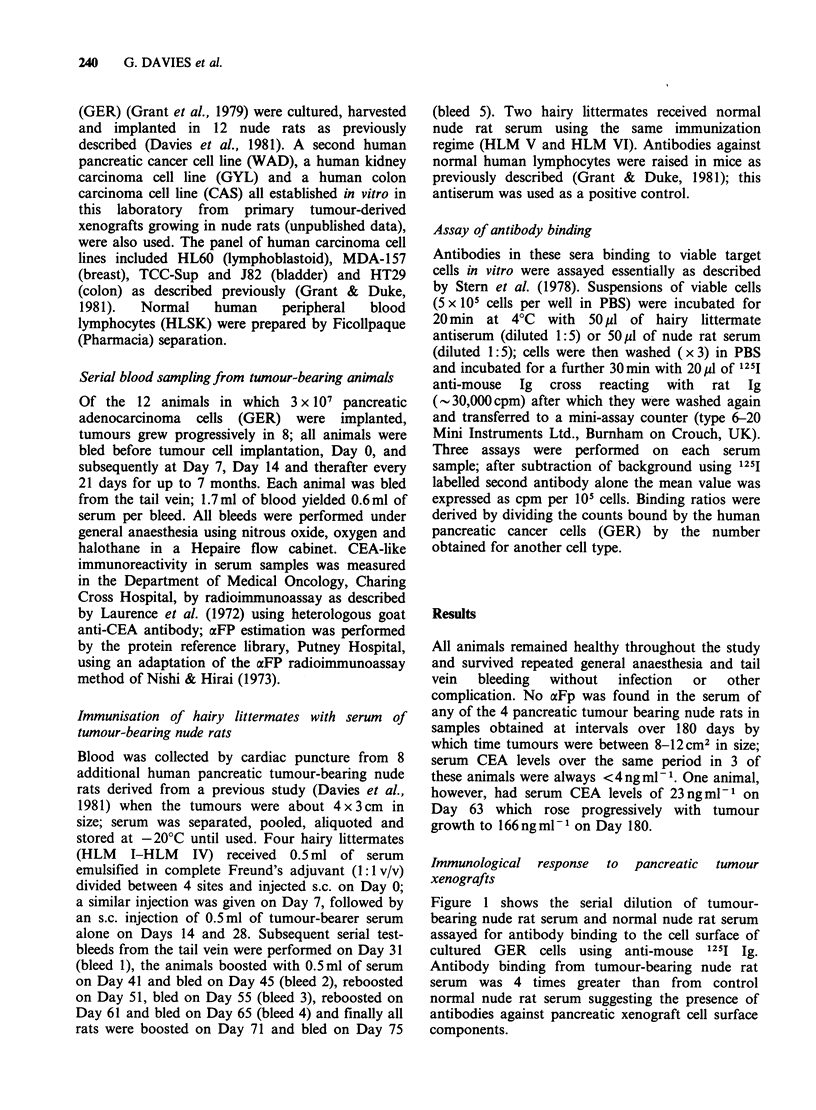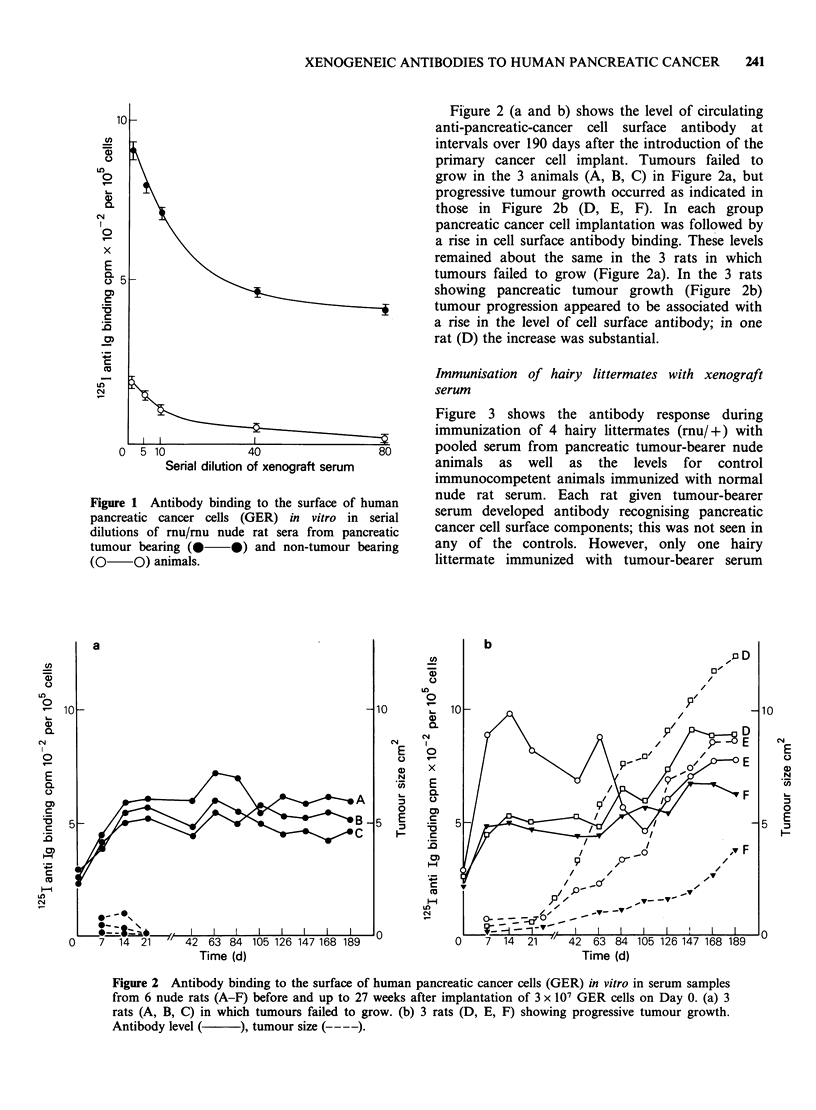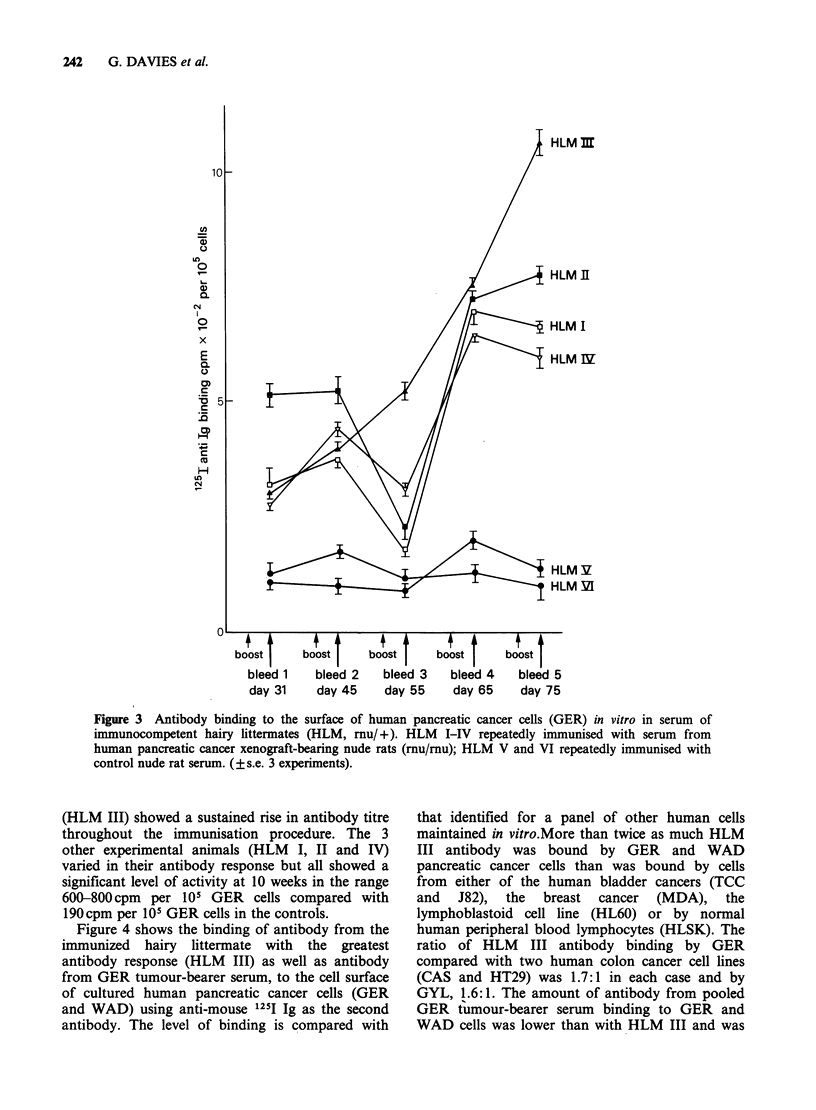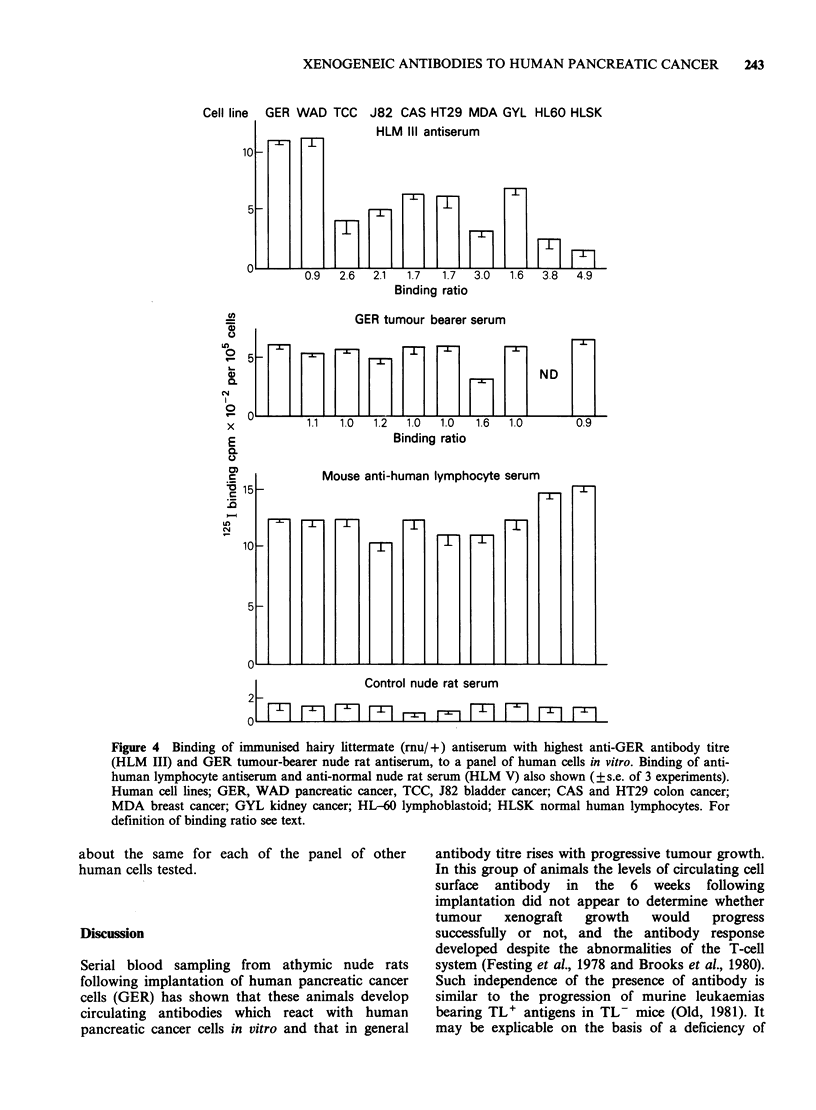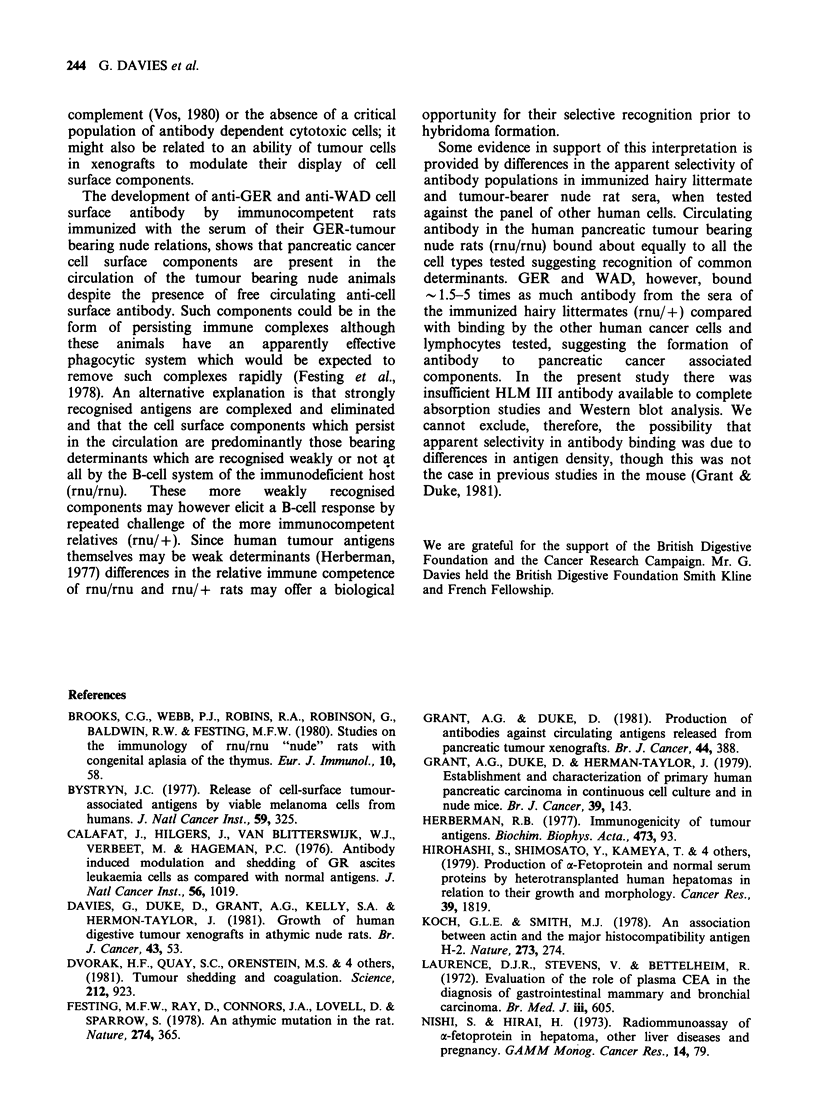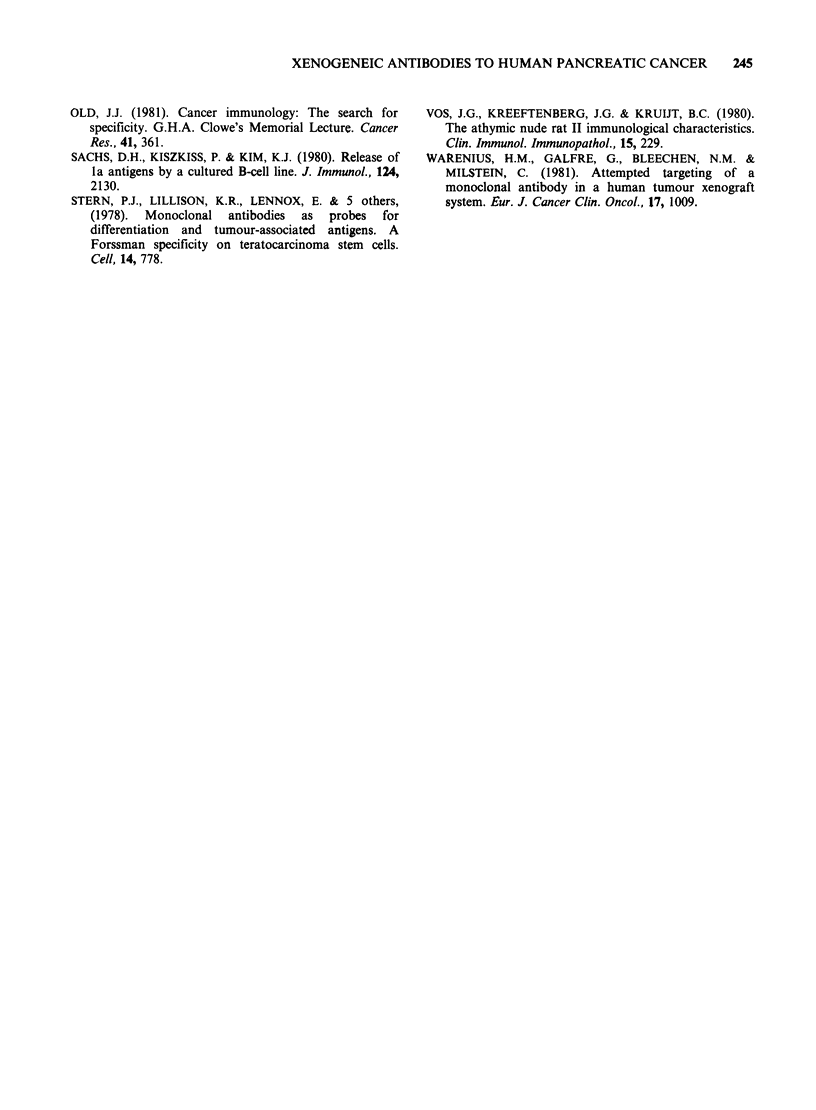# Antibody response of nude (RNU/RNU) and hairy (RNU/+) rats to circulating cell surface components from human pancreatic cancer xenografts.

**DOI:** 10.1038/bjc.1983.179

**Published:** 1983-08

**Authors:** G. Davies, A. G. Grant, D. Duke, J. Hermon-Taylor

## Abstract

Homozygous nude rats (rnu/rnu) injected s.c. with 3 X 10(7) human pancreatic cancer cells from the GER cell line developed circulating antibody to GER cell surface, detected in a 125I binding assay against viable GER cells in vitro. Antibody titre rose with progressive xenograft growth. These antibodies showed no selectivity for GER cells when compared with a panel of other human cell lines. Heterozygous nude rats (rnu/+) immunised with serum from their GER xenograft-bearing nude relatives (rnu/rnu) also developed anti-GER cell surface antibodies. These antibodies showed some selectivity for GER and WAD (a second human pancreatic cancer cell line) when compared with other human cancer cells and lymphocytes. These findings show that some human pancreatic cancer cell surface components may persist independently in the circulation of xenograft bearing rnu/rnu rats despite the presence of antibody excess to other surface determinants from the same cells. It is suggested that differences in the relative immune competence of rnu/rnu and rnu/+ rats may offer a biological opportunity for enhancing the recognition of weak antigenic determinants which may have some useful selectivity for different types of human tumour cells.


*Br. J. Cancer* (1983), **48**, 239–245

### Antibody response of nude (RNU/RNU) and hairy (RNU/+) rats to circulating cell surface components from human pancreatic cancer xenografts

G. Davies, A.G. Grant, D. Duke[1] & J. Hermon-Taylor

*Department of Surgery, St. George's Hospital Medical School, Cranmer Terrace, London, SW17 0RE and [1]Cancer Chemotherapy Department, Imperial Cancer Research Fund, Lincoln's Inn Field, London, WC2A 3PX.*

**Summary** Homozygous nude rats (rnu/rnu) injected s.c. with $3 \times 10^7$ human pancreatic cancer cells from the GER cell line developed circulating antibody to GER cell surface, detected in a $^{125}$I binding assay against viable GER cells *in vitro*. Antibody titre rose with progressive xenograft growth. These antibodies showed no selectivity for GER cells when compared with a panel of other human cell lines. Heterozygous nude rats (rnu/+) immunised with serum from their GER xenograft-bearing nude relatives (rnu/rnu) also developed anti-GER cell surface antibodies. These antibodies showed some selectivity for GER and WAD (a second human pancreatic cancer cell line) when compared with other human cancer cells and lymphocytes. These findings show that some human pancreatic cancer cell surface components may persist independently in the circulation of xenograft bearing rnu/rnu rats despite the presence of antibody excess to other surface determinants from the same cells. It is suggested that differences in the relative immune competence of rnu/rnu and rnu/+rats may offer a biological opportunity for enhancing the recognition of weak antigenic determinants which may have some useful selectivity for different types of human tumour cells.

Cell surface components have been shown to be released into tissue culture supernatants by human and animal tumour cells *in vitro* (Bystryn, 1977; Koch & Smith, 1978; Sachs *et al.*, 1980). There is evidence with animal tumours that this process may also occur in the host during tumour growth *in vivo* (Calafat *et al.*, 1976; Dvorak *et al.*, 1981; Warenius *et al.*, 1981). If this phenomenon is also a feature of human tumour growth in nude animals it may offer a biological selection which can be exploited in the production of monoclonal antibodies with some selectivity for the cancer cell surface. Immunocompetent mice immunised with serum from human tumour-bearing nude (nu/nu) relatives develop antibodies which have been shown to react with the surface of human tumour cells cultured *in vitro* (Grant & Duke, 1981).

Although circulating levels of alpha-fetoprotein ($\alpha$FP) have been estimated in repeated serum samples during human tumour growth in nude mice (Hirohashi *et al.*, 1979) the scope of such serial studies in the mouse is in general limited by the small blood volume of the animal. The athymic Rowett nude rat (rnu/rnu) has been shown to be a suitable animal for the *in vivo* maintenance of human pancreatic and colonic cancers both from primary surgical explants and from human pancreatic excorine adenocarcinoma cells (GER) in culture (Davies *et al.*, 1981). This larger nude mutant offers some experimental advantages which may complement the study of human tumours in nude mice.

The purpose of the present investigation was to examine the appearance of human pancreatic cancer cell-surface components in the circulation of the tumour-bearing nude rats by the antibody response of immunocompetent hairy littermates immunised with tumour-bearer sera. In addition we wished to characterise the antibody response of the tumour-bearer animal itself, to cell surface components throughout the period of pancreatic tumour growth.

### Materials and methods

### *Rats*

Three month old congenitally athymic female nude rats (rnu/rnu) were obtained from Olac 1976 Ltd. Hairy littermates (rnu/+) were bred in our laboratory by mating homozygous nude males (rnu/rnu) with heterozygous females (rnu/+). All the rats used in this study were maintained in an isolated facility; nude animals were protected in filter boxes and their immunocompetent hairy littermates were housed in conventional conditions.

### *Cell lines and xenografts*

Human pancreatic exocrine adenocarcinoma cells

Correspondence: J. Hermon-Taylor
Received 3 February 1983; accepted 26 April 1983.

(GER) (Grant et al., 1979) were cultured, harvested and implanted in 12 nude rats as previously described (Davies et al., 1981). A second human pancreatic cancer cell line (WAD), a human kidney carcinoma cell line (GYL) and a human colon carcinoma cell line (CAS) all established *in vitro* in this laboratory from primary tumour-derived xenografts growing in nude rats (unpublished data), were also used. The panel of human carcinoma cell lines included HL60 (lymphoblastoid), MDA-157 (breast), TCC-Sup and J82 (bladder) and HT29 (colon) as described previously (Grant & Duke, 1981). Normal human peripheral blood lymphocytes (HLSK) were prepared by Ficollpaque (Pharmacia) separation.

*Serial blood sampling from tumour-bearing animals*

Of the 12 animals in which $3 \times 10^7$ pancreatic adenocarcinoma cells (GER) were implanted, tumours grew progressively in 8; all animals were bled before tumour cell implantation, Day 0, and subsequently at Day 7, Day 14 and therafter every 21 days for up to 7 months. Each animal was bled from the tail vein; 1.7 ml of blood yielded 0.6 ml of serum per bleed. All bleeds were performed under general anaesthesia using nitrous oxide, oxygen and halothane in a Hepaire flow cabinet. CEA-like immunoreactivity in serum samples was measured in the Department of Medical Oncology, Charing Cross Hospital, by radioimmunoassay as described by Laurence et al. (1972) using heterologous goat anti-CEA antibody; αFP estimation was performed by the protein reference library, Putney Hospital, using an adaptation of the αFP radioimmunoassay method of Nishi & Hirai (1973).

*Immunisation of hairy littermates with serum of tumour-bearing nude rats*

Blood was collected by cardiac puncture from 8 additional human pancreatic tumour-bearing nude rats derived from a previous study (Davies et al., 1981) when the tumours were about 4 × 3 cm in size; serum was separated, pooled, aliquoted and stored at −20°C until used. Four hairy littermates (HLM I–HLM IV) received 0.5 ml of serum emulsified in complete Freund's adjuvant (1:1 v/v) divided between 4 sites and injected s.c. on Day 0; a similar injection was given on Day 7, followed by an s.c. injection of 0.5 ml of tumour-bearer serum alone on Days 14 and 28. Subsequent serial test-bleeds from the tail vein were performed on Day 31 (bleed 1), the animals boosted with 0.5 ml of serum on Day 41 and bled on Day 45 (bleed 2), reboosted on Day 51, bled on Day 55 (bleed 3), reboosted on Day 61 and bled on Day 65 (bleed 4) and finally all rats were boosted on Day 71 and bled on Day 75 (bleed 5). Two hairy littermates received normal nude rat serum using the same immunization regime (HLM V and HLM VI). Antibodies against normal human lymphocytes were raised in mice as previously described (Grant & Duke, 1981); this antiserum was used as a positive control.

*Assay of antibody binding*

Antibodies in these sera binding to viable target cells *in vitro* were assayed essentially as described by Stern et al. (1978). Suspensions of viable cells ($5 \times 10^5$ cells per well in PBS) were incubated for 20 min at 4°C with 50 μl of hairy littermate antiserum (diluted 1:5) or 50 μl of nude rat serum (diluted 1:5); cells were then washed (×3) in PBS and incubated for a further 30 min with 20 μl of $^{125}$I anti-mouse Ig cross reacting with rat Ig (~30,000 cpm) after which they were washed again and transferred to a mini-assay counter (type 6–20 Mini Instruments Ltd., Burnham on Crouch, UK). Three assays were performed on each serum sample; after subtraction of background using $^{125}$I labelled second antibody alone the mean value was expressed as cpm per $10^5$ cells. Binding ratios were derived by dividing the counts bound by the human pancreatic cancer cells (GER) by the number obtained for another cell type.

### Results

All animals remained healthy throughout the study and survived repeated general anaesthesia and tail vein bleeding without infection or other complication. No αFp was found in the serum of any of the 4 pancreatic tumour bearing nude rats in samples obtained at intervals over 180 days by which time tumours were between 8–12 cm$^2$ in size; serum CEA levels over the same period in 3 of these animals were always $<4$ ng ml$^{-1}$. One animal, however, had serum CEA levels of 23 ng ml$^{-1}$ on Day 63 which rose progressively with tumour growth to 166 ng ml$^{-1}$ on Day 180.

*Immunological response to pancreatic tumour xenografts*

Figure 1 shows the serial dilution of tumour-bearing nude rat serum and normal nude rat serum assayed for antibody binding to the cell surface of cultured GER cells using anti-mouse $^{125}$I Ig. Antibody binding from tumour-bearing nude rat serum was 4 times greater than from control normal nude rat serum suggesting the presence of antibodies against pancreatic xenograft cell surface components.

**Figure 1** Antibody binding to the surface of human pancreatic cancer cells (GER) *in vitro* in serial dilutions of rnu/rnu nude rat sera from pancreatic tumour bearing (●——●) and non-tumour bearing (○——○) animals.

Figure 2 (a and b) shows the level of circulating anti-pancreatic-cancer cell surface antibody at intervals over 190 days after the introduction of the primary cancer cell implant. Tumours failed to grow in the 3 animals (A, B, C) in Figure 2a, but progressive tumour growth occurred as indicated in those in Figure 2b (D, E, F). In each group pancreatic cancer cell implantation was followed by a rise in cell surface antibody binding. These levels remained about the same in the 3 rats in which tumours failed to grow (Figure 2a). In the 3 rats showing pancreatic tumour growth (Figure 2b) tumour progression appeared to be associated with a rise in the level of cell surface antibody; in one rat (D) the increase was substantial.

*Immunisation of hairy littermates with xenograft serum*

Figure 3 shows the antibody response during immunization of 4 hairy littermates (rnu/+) with pooled serum from pancreatic tumour-bearer nude animals as well as the levels for control immunocompetent animals immunized with normal nude rat serum. Each rat given tumour-bearer serum developed antibody recognising pancreatic cancer cell surface components; this was not seen in any of the controls. However, only one hairy littermate immunized with tumour-bearer serum

**Figure 2** Antibody binding to the surface of human pancreatic cancer cells (GER) *in vitro* in serum samples from 6 nude rats (A–F) before and up to 27 weeks after implantation of $3\times10^7$ GER cells on Day 0. (a) 3 rats (A, B, C) in which tumours failed to grow. (b) 3 rats (D, E, F) showing progressive tumour growth. Antibody level (———), tumour size (- - - -).

**Figure 3** Antibody binding to the surface of human pancreatic cancer cells (GER) *in vitro* in serum of immunocompetent hairy littermates (HLM, rnu/+). HLM I–IV repeatedly immunised with serum from human pancreatic cancer xenograft-bearing nude rats (rnu/rnu); HLM V and VI repeatedly immunised with control nude rat serum. (±s.e. 3 experiments).

(HLM III) showed a sustained rise in antibody titre throughout the immunisation procedure. The 3 other experimental animals (HLM I, II and IV) varied in their antibody response but all showed a significant level of activity at 10 weeks in the range 600–800 cpm per $10^5$ GER cells compared with 190 cpm per $10^5$ GER cells in the controls.

Figure 4 shows the binding of antibody from the immunized hairy littermate with the greatest antibody response (HLM III) as well as antibody from GER tumour-bearer serum, to the cell surface of cultured human pancreatic cancer cells (GER and WAD) using anti-mouse $^{125}$I Ig as the second antibody. The level of binding is compared with that identified for a panel of other human cells maintained *in vitro*.More than twice as much HLM III antibody was bound by GER and WAD pancreatic cancer cells than was bound by cells from either of the human bladder cancers (TCC and J82), the breast cancer (MDA), the lymphoblastoid cell line (HL60) or by normal human peripheral blood lymphocytes (HLSK). The ratio of HLM III antibody binding by GER compared with two human colon cancer cell lines (CAS and HT29) was 1.7:1 in each case and by GYL, 1.6:1. The amount of antibody from pooled GER tumour-bearer serum binding to GER and WAD cells was lower than with HLM III and was

**Figure 4** Binding of immunised hairy littermate (rnu/+) antiserum with highest anti-GER antibody titre (HLM III) and GER tumour-bearer nude rat antiserum, to a panel of human cells *in vitro*. Binding of anti-human lymphocyte antiserum and anti-normal nude rat serum (HLM V) also shown (±s.e. of 3 experiments). Human cell lines; GER, WAD pancreatic cancer, TCC, J82 bladder cancer; CAS and HT29 colon cancer; MDA breast cancer; GYL kidney cancer; HL–60 lymphoblastoid; HLSK normal human lymphocytes. For definition of binding ratio see text.

about the same for each of the panel of other human cells tested.

### Discussion

Serial blood sampling from athymic nude rats following implantation of human pancreatic cancer cells (GER) has shown that these animals develop circulating antibodies which react with human pancreatic cancer cells *in vitro* and that in general antibody titre rises with progressive tumour growth. In this group of animals the levels of circulating cell surface antibody in the 6 weeks following implantation did not appear to determine whether tumour xenograft growth would progress successfully or not, and the antibody response developed despite the abnormalities of the T-cell system (Festing *et al.*, 1978 and Brooks *et al.*, 1980). Such independence of the presence of antibody is similar to the progression of murine leukaemias bearing $TL^+$ antigens in $TL^-$ mice (Old, 1981). It may be explicable on the basis of a deficiency of complement (Vos, 1980) or the absence of a critical population of antibody dependent cytotoxic cells; it might also be related to an ability of tumour cells in xenografts to modulate their display of cell surface components.

The development of anti-GER and anti-WAD cell surface antibody by immunocompetent rats immunized with the serum of their GER-tumour bearing nude relations, shows that pancreatic cancer cell surface components are present in the circulation of the tumour bearing nude animals despite the presence of free circulating anti-cell surface antibody. Such components could be in the form of persisting immune complexes although these animals have an apparently effective phagocytic system which would be expected to remove such complexes rapidly (Festing et al., 1978). An alternative explanation is that strongly recognised antigens are complexed and eliminated and that the cell surface components which persist in the circulation are predominantly those bearing determinants which are recognised weakly or not at all by the B-cell system of the immunodeficient host (rnu/rnu). These more weakly recognised components may however elicit a B-cell response by repeated challenge of the more immunocompetent relatives (rnu/+). Since human tumour antigens themselves may be weak determinants (Herberman, 1977) differences in the relative immune competence of rnu/rnu and rnu/+ rats may offer a biological opportunity for their selective recognition prior to hybridoma formation.

Some evidence in support of this interpretation is provided by differences in the apparent selectivity of antibody populations in immunized hairy littermate and tumour-bearer nude rat sera, when tested against the panel of other human cells. Circulating antibody in the human pancreatic tumour bearing nude rats (rnu/rnu) bound about equally to all the cell types tested suggesting recognition of common determinants. GER and WAD, however, bound ~1.5–5 times as much antibody from the sera of the immunized hairy littermates (rnu/+) compared with binding by the other human cancer cells and lymphocytes tested, suggesting the formation of antibody to pancreatic cancer associated components. In the present study there was insufficient HLM III antibody available to complete absorption studies and Western blot analysis. We cannot exclude, therefore, the possibility that apparent selectivity in antibody binding was due to differences in antigen density, though this was not the case in previous studies in the mouse (Grant & Duke, 1981).

We are grateful for the support of the British Digestive Foundation and the Cancer Research Campaign. Mr. G. Davies held the British Digestive Foundation Smith Kline and French Fellowship.

### References

BROOKS, C.G., WEBB, P.J., ROBINS, R.A., ROBINSON, G., BALDWIN, R.W. & FESTING, M.F.W. (1980). Studies on the immunology of rnu/rnu "nude" rats with congenital aplasia of the thymus. *Eur. J. Immunol.*, **10**, 58.

BYSTRYN, J.C. (1977). Release of cell-surface tumour-associated antigens by viable melanoma cells from humans. *J. Natl Cancer Inst.*, **59**, 325.

CALAFAT, J., HILGERS, J., VAN BLITTERSWIJK, W.J., VERBEET, M. & HAGEMAN, P.C. (1976). Antibody induced modulation and shedding of GR ascites leukaemia cells as compared with normal antigens. *J. Natl Cancer Inst.*, **56**, 1019.

DAVIES, G., DUKE, D., GRANT, A.G., KELLY, S.A. & HERMON-TAYLOR, J. (1981). Growth of human digestive tumour xenografts in athymic nude rats. *Br. J. Cancer*, **43**, 53.

DVORAK, H.F., QUAY, S.C., ORENSTEIN, M.S. & 4 others, (1981). Tumour shedding and coagulation. *Science*, **212**, 923.

FESTING, M.F.W., RAY, D., CONNORS, J.A., LOVELL, D. & SPARROW, S. (1978). An athymic mutation in the rat. *Nature*, **274**, 365.

GRANT, A.G. & DUKE, D. (1981). Production of antibodies against circulating antigens released from pancreatic tumour xenografts. *Br. J. Cancer*, **44**, 388.

GRANT, A.G., DUKE, D. & HERMAN-TAYLOR, J. (1979). Establishment and characterization of primary human pancreatic carcinoma in continuous cell culture and in nude mice. *Br. J. Cancer*, **39**, 143.

HERBERMAN, R.B. (1977). Immunogenicity of tumour antigens. *Biochim. Biophys. Acta.*, **473**, 93.

HIROHASHI, S., SHIMOSATO, Y., KAMEYA, T. & 4 others, (1979). Production of α-Fetoprotein and normal serum proteins by heterotransplanted human hepatomas in relation to their growth and morphology. *Cancer Res.*, **39**, 1819.

KOCH, G.L.E. & SMITH, M.J. (1978). An association between actin and the major histocompatibility antigen H-2. *Nature*, **273**, 274.

LAURENCE, D.J.R., STEVENS, V. & BETTELHEIM, R. (1972). Evaluation of the role of plasma CEA in the diagnosis of gastrointestinal mammary and bronchial carcinoma. *Br. Med. J.* **iii**, 605.

NISHI, S. & HIRAI, H. (1973). Radiommunoassay of α-fetoprotein in hepatoma, other liver diseases and pregnancy. *GAMM Monog. Cancer Res.*, **14**, 79.

OLD, J.J. (1981). Cancer immunology: The search for specificity. G.H.A. Clowe's Memorial Lecture. *Cancer Res.*, **41**, 361.

SACHS, D.H., KISZKISS, P. & KIM, K.J. (1980). Release of la antigens by a cultured B-cell line. *J. Immunol.*, **124**, 2130.

STERN, P.J., LILLISON, K.R., LENNOX, E. & 5 others, (1978). Monoclonal antibodies as probes for differentiation and tumour-associated antigens. A Forssman specificity on teratocarcinoma stem cells. *Cell*, **14**, 778.

VOS, J.G., KREEFTENBERG, J.G. & KRUIJT, B.C. (1980). The athymic nude rat II immunological characteristics. *Clin. Immunol. Immunopathol.*, **15**, 229.

WARENIUS, H.M., GALFRE, G., BLEECHEN, N.M. & MILSTEIN, C. (1981). Attempted targeting of a monoclonal antibody in a human tumour xenograft system. *Eur. J. Cancer Clin. Oncol.*, **17**, 1009.